# Assessing compliance with national guidelines in diabetes care: A study leveraging data from south Africa’s National Health Laboratory Service (NHLS)

**DOI:** 10.1371/journal.pgph.0003014

**Published:** 2024-09-03

**Authors:** A. T. Brennan, E. M. Kileel, M. P. Fox, J. A. George, S Khoza, S. Rosen, F. Raal, P. Hibberd, K. Chetty, K. Mlisana, J. Bor, N. J. Crowther

**Affiliations:** 1 Faculty of Health Sciences University of the Witwatersrand, Health Economics and Epidemiology Research Office, Johannesburg, South Africa; 2 Department of Global Health, Boston University School of Public Health, Boston, Massachusetts, United States of America; 3 Department of Epidemiology, Boston University School of Public Health, Boston, Massachusetts, United States of America; 4 Wits Diagnostic Innovation Hub, University of the Witwatersrand, Johannesburg, South Africa; 5 National Health Laboratory Service, Johannesburg, Gauteng, South Africa; 6 Faculty of Health Sciences, Department of Chemical Pathology, University of the Witwatersrand, Johannesburg, South Africa; 7 Faculty of Health Sciences, Department of Internal Medicine, Division of Endocrinology and Metabolism, University of the Witwatersrand, Johannesburg, South Africa; 8 Academic Affairs, Research and Quality Assurance, National Health Laboratory Service, Johannesburg, South Africa; University of Embu, KENYA

## Abstract

Diabetes is a major global health issue. We evaluated compliance to laboratory-based management guidelines for diabetes (type 1 and 2), essential for effective treatment and reducing diabetes-related morbidity and mortality. Our study utilized South Africa’s National Health Laboratory Services (NHLS) data, focusing on patients from birth to age 80 years who underwent initial diabetes laboratory testing between January 1, 2012-January 1, 2016. Patients were categorized into type 1 (<30 years) or type 2 (≥30–80 years) diabetes based on age at first diabetes test. National diabetes guidelines recommend blood glucose to be checked every three-six months post laboratory-diagnosis. We employed a sharp regression discontinuity design to estimate the effect of a laboratory-diagnosis of diabetes on the likelihood of having a follow-up laboratory test 24 months post-diagnosis. Among patients with type 2 diabetes, the probability of a diabetes follow-up laboratory test within 24 months was 52.4% for patients presenting above the diabetes diagnosis threshold vs 31.1% for those presenting below. Although the likelihood of repeat testing rose with higher HbA1c and glucose levels, at the diagnostic threshold there was no clinically meaningful difference (risk difference: -2.2%, 95% CI: -3.3%, -1.2%). These results were consistent among patients with type 1 diabetes, those living with and without HIV, and healthcare setting. In a national laboratory cohort, diabetes laboratory-diagnosis did not lead to increased monitoring as recommended in national guidelines. Strategies to improve patient education, healthcare provider communication, and healthcare system support are essential to enhance guideline compliance and overall diabetes management.

## Introduction

The global prevalence of both type 1 and type 2 diabetes is increasing. It is estimated that nearly 537 million adults are living with diabetes worldwide, with 90% of diagnoses occurring in low- and middle-income countries (LMICs) [1]. While data on the prevalence of type 1 diabetes in South Africa is lacking, the estimated prevalence of type 2 diabetes has more than doubled in the last decade, with recent data suggesting that approximately 13% of South African adults are living with type 2 diabetes [2]. This trend is primarily ascribed to urbanization and changes in dietary habits and physical inactivity [2] and is particularly concerning in countries like South Africa, which accounts for approximately 19% of the world’s HIV cases [3], thereby creating a complex multimorbidity disease environment.

In response to the growing trend of noncommunicable diseases, like diabetes, the World Health Organization (WHO) introduced the *Global action plan for the prevention and control of noncommunicable diseases* with the goal of reducing and preventing morbidity, mortality, and disability of noncommunicable diseases [4]. Specifically, the WHO aims to halt the rise in diabetes and achieve a 25% relative reduction in overall mortality from cardiovascular disease, respiratory disease, cancer, or diabetes [4]. As a tool to achieve these targets, the plan references the use of guidelines in primary health care settings for the diagnosis and management of type 2 diabetes [4].

According to most guidelines, a diagnosis of diabetes should be made if a patient’s laboratory-determined fasting blood glucose is ≥7.0 mmol/L, random blood glucose is ≥11.1 mmol/L, or glycated hemoglobin A1c (HbA1c) is ≥6.5% and symptoms of hyperglycemia are present [5]. After diagnosis, if a patient achieves glycemic control with unchanged treatment, it is recommended they undergo formal laboratory blood glucose monitoring every six months. Conversely, if glycemic control is not achieved or if treatment is modified or intensified, blood glucose should be monitored every three months [5,6].

Management of diabetes continues to be a multifaceted challenge [7], and when left undetected or inadequately managed, can result in serious and potentially fatal complications, including heart disease, kidney failure, or blindness [8,9]. Rigorous compliance to diabetes management guidelines by both clinicians and patients, as well as systematic tracking of blood glucose levels through regular consultations, is critical in averting complications, decreasing mortality rates, and curtailing healthcare expenditures [9].

In this analysis, we sought to evaluate compliance (i.e., following the guidelines as they are stated) to diabetes management guidelines using data from South Africa’s National Health Laboratory Service (NHLS) database, applying a regression discontinuity approach. We hypothesized that a laboratory test above the diagnostic threshold for diabetes would result in a sharp increase in the likelihood of having a diabetes follow-up laboratory test of glycemia, in accordance with national guidelines [5]. This examination underscores the importance of comprehensive follow-up care, emphasizing the synergy between early diagnosis, guideline-based treatment, and the prevention of debilitating complications.

## Methods

### Data source

South Africa’s NHLS is the sole provider of laboratory services for the public sector health system, which serves 80% of the population across all provinces [10]. Due to differences in reporting and recording of patient information associated with laboratory tests, a single patient may be represented by several different sets of identifying information in the NHLS database. In collaboration with the NHLS, CD4 and HIV viral load patient laboratory tests were linked using a graph-based probabilistic record linkage approach to create a validated, anonymous patient identifier. This process has been reported previously [11]. Briefly, a graph-based probabilistic algorithm was applied using Jaro-Winkler string comparisons [12] and customized matching thresholds devised to link together all labs for a single patient within the national HIV program. The matching process was informed by available demographic data for each specimen, including first name, last name, date of birth, gender, province, and medical facility. The algorithm was developed and validated on 59,000 potential matches from a sample of 1,000 specimens for application and attained a sensitivity of 93.7% and positive predictive values (PPV) of 98.6% [11]. The record-linking methods were extended to all HIV, TB, and non-communicable disease (NCD) laboratory tests in 2019 to create the NHLS Multi-morbidity Cohort used for this analysis.

Currently, the NHLS Multi-morbidity Cohort has over 68 million laboratory measurements corresponding to over 49 million unique patients from birth with at least one laboratory measurement between April 1, 2004, and March 31, 2017. It contains a unique anonymized patient identifier, biological sex, age, laboratory test date, test type, test result, health facility, district, and province.

### Diabetes guidelines

During the study period, the 2012 Society for Endocrinology, Metabolism, and Diabetes of South Africa (SEMDSA) guidelines [5] were the screening, diagnosis, and treatment guidelines meant to guide public-sector diabetes care throughout South Africa. The guidelines recommended diabetes screening in low-risk individuals who undergo glucose testing incidentally (random screening), in those identified as high-risk during consultations for unrelated health matters (opportunistic screening), and in those deliberately tested because of their high-risk status and symptomology (targeted screening) [5]. The guidelines endorsed use of glycated hemoglobin A1c (HbA1c) for screening and diagnosing diabetes [5], in addition to random and fasting blood glucose tests. In asymptomatic individuals, an oral glucose tolerance test (OGTT) can be performed or HbA1c measured. If fasting blood glucose is ≥7.0 mmol/L or 2-hour post-glucose load blood glucose is ≥11.1 mmol/L, or hemoglobin A1C (HbA1c) is ≥6.5% and any of these measurements are confirmed at a repeat test within 2 weeks of the first, a diagnosis of diabetes can be made.

Guidelines required diagnosis of diabetes be based on formal laboratory testing and not point-of-care instruments [5]. After a diagnosis of type 1 or type 2 diabetes, if a patient achieves glycemic control with unchanged treatment, it is recommended to undergo formal laboratory blood glucose monitoring every six months. Conversely, if glycemic control is not achieved or if treatment is modified or intensified, blood glucose should be monitored every three months [5]. All laboratory tests used to diagnose diabetes in South Africa’s public sector health system are contained within the NHLS Cohort.

### Study design

We conducted a prospective cohort study using a regression discontinuity design. The study sample included patients from birth to age 80 years who had their first diabetes laboratory test (HbA1c, fasting plasma glucose or random plasma glucose) between January 1, 2012, and January 1, 2016. All patients had the potential for at least 24-months of follow-up after their initial diabetes test.

Given the absence of further clinical or laboratory information to distinguish between diabetes types, we relied on the surrogate of age at the first diabetes test to differentiate between type 1 and type 2 diabetes. Patients diagnosed with diabetes through laboratory tests between the ages of 0 and <30 years were categorized as having type 1 diabetes, while patients with a laboratory diagnosis of diabetes between the ages of 30 and 80 years were classified as having type 2 diabetes. Additionally, we stratified the analysis by HIV status determined by the presence of a CD4 or viral load test any time before their first diabetes test, or up to 24-months after, as guidelines specify that all persons testing positive for HIV should have a CD4 count. If neither a CD4 nor viral load test was present in the database, patients were considered HIV-uninfected.

### Exposure

The primary exposure was a laboratory test result indicative of diabetes, defined according to the 2012 SEMDSA guidelines that were in effect during the study period: random plasma glucose ≥11.1 mmol/L, fasting plasma glucose ≥7.0 mmol/L, or HbA1c ≥6.5% [5].

To increase statistical power, we combined all three diagnostic measures by standardizing diabetes test results into a Z-score. The diabetes Z-score measure was calculated by taking the difference between a participants’ diabetes test result and the respective diagnosis threshold and dividing by the standard deviation of the diabetes test result—see formula 1 below:

$$Diabetes\;Zscore=\frac{\left(Diabetes\;test\;result-Threshold\;value\right)}{Population\;standard\;deviation\;of\;diabetes\;test\;result}$$

*where Threshold value is equal to 7.0 for fasting glucose, 11.1 for random glucose, and 6.5 for HbA1c.

A Z-score value greater than 1 corresponded to a test result greater than the diagnostic threshold. A Z-score value less than 0 corresponded to a test result less than the diagnostic threshold. Z-score values were then log-transformed to obtain a normal distribution. We removed extreme values by trimming above the 99^th^ and below the 1^st^ percentiles of the log-standardized test result.

### Outcome

Our primary outcome of interest was whether a participant had a diabetes follow-up laboratory test (HbA1c, fasting or random plasma glucose) within 24 months of their first diabetes test. We allowed a larger window of time to capture any potential variations in follow-up patterns.

### Statistical analysis

**Fig 1:**
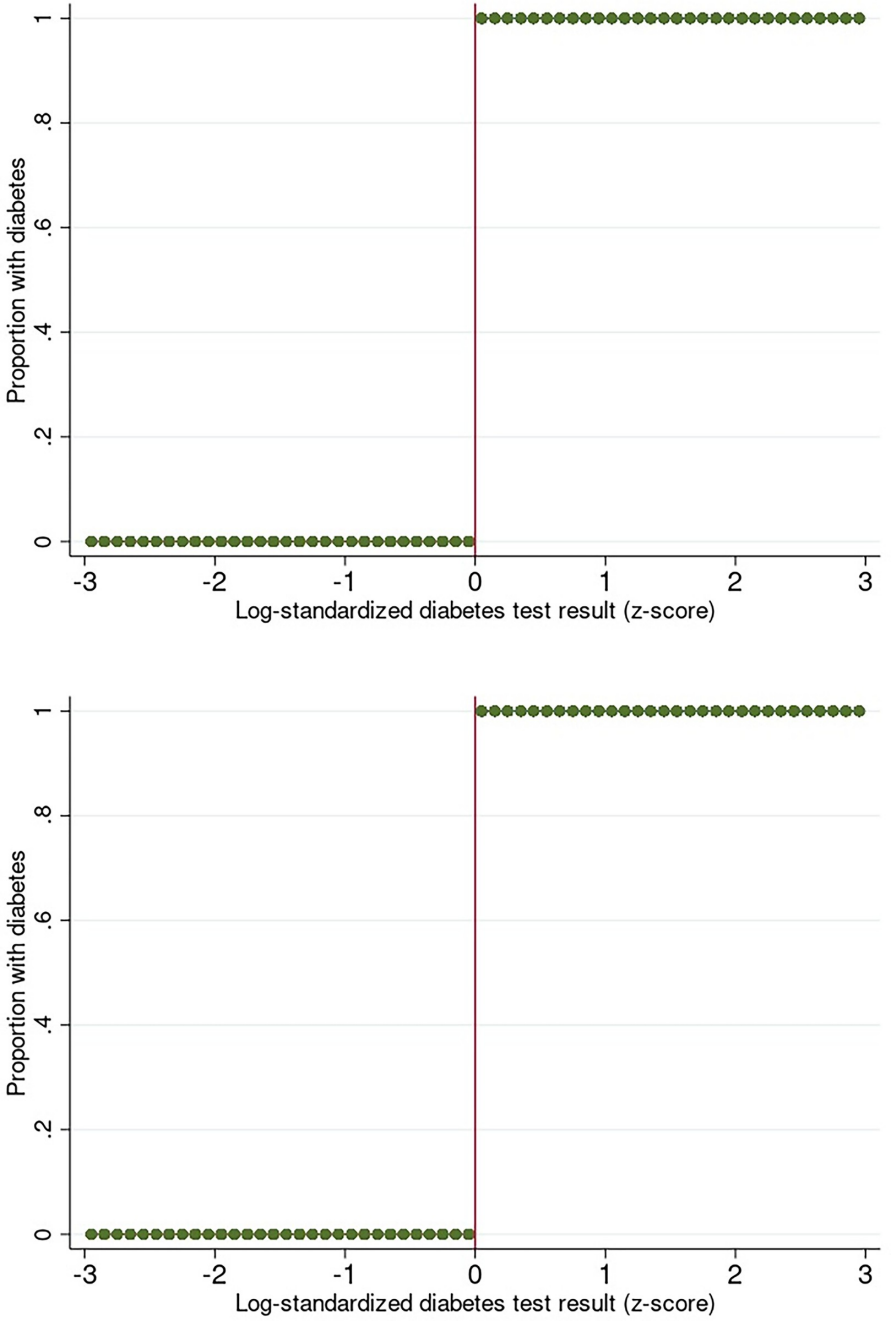
Proportion of patients with a log-standardized laboratory diagnosis of diabetes above and below the diagnostic threshold among patients a) in the type 2 diabetes cohort and b) in the type 1 diabetes cohort.

We used a sharp regression discontinuity design [13,14] to evaluate whether a laboratory test result indicative of diabetes resulted in an increased likelihood of having a follow-up test of glycemia in the proceeding 24-months. The regression discontinuity design is a quasi-experimental method that can be used to estimate causal effects of an intervention when the assignment variable is determined by a threshold rule [13,14]. As the variable that determined exposure status (diabetes test result) is continuous in nature, it is subject to random variability and as such offers a “natural experiment” comparing patients just above and just below the diagnostic threshold [13,14]. Patients were assigned to the treatment or control group based on the value of their diabetes test result. We created an indicator variable equal to 1 (treatment) if the patients log-transformed diabetes Z-score value was greater than 0, and equal to 0 (control) otherwise (Fig 1). As long as neither patients nor clinicians manipulated diabetes test results, the random fluctuation of diabetes test results around the threshold should mean that patients falling immediately above and below this mark are comparable in both observed and unobserved characteristics [13].

Using this approach, we estimated the intention-to-treat (ITT) effects of obtaining a laboratory value indicative of diabetes on the likelihood of having a follow-up test of glycemia within 24 months. Our ITT effect was identified as the intercept shift at a Z-score of zero (i.e., the coefficient on the threshold indicator). This estimation was conducted on the risk difference scale using local polynomial regression discontinuity with robust confidence intervals and inference procedures, employing Stata’s rdrobust tool [15]. In a regression discontinuity design, effect estimates are estimated within a bandwidth around the threshold to achieve local randomization [13,14]. The optimal bandwidth for the analysis was determined through the rdbwselect function in Stata [16]. We controlled for continuous linear trends in outcomes over Z-scores, allowing for different slopes before and after the threshold. Scatterplots were overlaid onto linear regression plots, binned in Z-score intervals of 0.1, to display the average outcomes in each segment. The analyses were conducted using Stata v16.0.

### Assessing manipulation of the assignment variable

To ensure patients on either side of the threshold were exchangeable, we assessed continuity of observed baseline covariates (e.g., age, biological sex, testing location (primary health care (PHC) facility or hospital setting) and amongst patients living with HIV and without HIV) using regression models similar to the ITT, where instead of regressing the outcome on the model parameters we regressed each observable variable separately. The coefficient on the intercept represented the mean value just below the threshold (where indicator was equal to 0), and the coefficient on the intercept plus the estimate on the threshold indicator represented the mean value just above the threshold (where indicator was equal to 1). Further, the McCrary Density test was used to assess for bunching or manipulation of the assignment variable on either side of the diagnostic threshold [17]. For the purposes of evaluating modeling assumptions, meaningful differences and bunching was based on an alpha value of 0.05. So long as these assumptions were met, we concluded that patients on either side of the threshold were exchangeable on observed and unobserved characteristics, allowing us to establish causal associations between receiving a diabetes diagnosis and the likelihood of having a follow-up laboratory test of glycemia.

### Robustness checks

To ensure the results were not influenced by the standardization of the diabetes test results, we ran each regression discontinuity on the crude data, i.e., not standardized or log-transformed, in both the type 1 and type 2 cohorts. As each test has a different diagnostic threshold, we ran separate regression discontinuity analyses for those who received a fasting glucose, random glucose, or HbA1c.

### Ethics approval and consent for participation

Approval for analysis of de-identified NHLS data was granted by Boston University’s Institutional Review Board (protocol no. H-41152), Human Research Ethics Committee of the University of the Witwatersrand (protocol no. M200851) and NHLS Academic Affairs and Research Management System (protocol no. PR2232386). Our research was observational, not experimental, in nature. We conducted our analysis using existing NHLS laboratory data from South Africa that is used for routine clinical care of patients in government sector health facilities. We had no contact with patients and no experiments were conducted on humans or human tissues, as such, informed consent was not obtained from subjects and/or their legal guardian(s). An inform consent waiver was received from the Boston University’s Institutional Review Board and the Human Research Ethics Committee of the University of the Witwatersrand.

## Results

### Study population

Over the follow-up period, a total of 826,977 patients underwent a diabetes laboratory test at a public health facility. Of these, 12% were administered a fasting glucose test, 26% a random glucose test, and 62% an HbA1c test (Table 1). The overall cohort’s median age was 53 years (interquartile range (IQR) of 37–65 years); the majority were female (64%), living without HIV (80%), and had their first diabetes test in a hospital setting (62%). Among the 695,729 patients aged 30–80 years (type 2 cohort), 50.6% had a laboratory test result indicative of diabetes. Of the 144,269 patients aged < 30 years, 15.1% had a test result indicative of diabetes. In a comparison between the type 2 and type 1 diabetes cohorts, aside from the anticipated difference in median age (55 years for type 2 vs. 22 years for type 1), the type 1 cohort displayed a marginally higher proportion of individuals living with HIV (19% for type 2 vs 25% for type 1) and a substantially larger proportion having their initial diabetes test in a hospital environment (58% for type 2 vs 82% for type 1).

**Table 1:** Demographic characteristics of type 2 and type 1 analysis cohorts.

	type 2 diabetes (n = 685,568; 82.9%)	type 1 diabetes (n = 141,409; 17.1%)	Total (n = 826,977)
**Test**			
Fasting glucose, n (%)	79,549 (12%)	20,267 (14%)	99,816 (12%)
Random glucose, n (%)	183,215 (27%)	32,110 (23%)	215,325 (26%)
HbA1c, n (%)	422,804 (62%)	89,032 (63%)	511,836 (62%)
**Age (years)**, median (q1, q3)	54 (44, 63)	22 (9, 26)	53 (37, 65)
**Biologic sex**			
Female, n (%)	441,919 (64%)	90,357 (64%)	532,276 (64%)
Male, n (%)	243,649 (36%)	51,052 (36%)	294,701 (36%)
**HIV status**			
People living with HIV, n (%)	131,057 (19%)	35,052 (25%)	166,109 (20%)
People living without HIV, n (%)	554,511 (81%)	106,357 (75%)	660,868 (80%)
**First test location**			
Hospital, n (%)	399,892 (58%)	116,063 (82%)	515,955 (62%)
Primary Health Centre, n (%)	255,252 (37%)	19,738 (14%)	274,990 (33%)
Missing	30,424 (4%)	5608 (4%)	36,032 (4%)
**Province**			
KwaZulu Natal	207,505 (30%)	37,929 (27%)	245,434 (30%)
Gauteng	183,732 (27%)	46,225 (33%)	229,957 (28%)
Eastern Cape	80,202 (12%)	13,239 (9%)	93,441 (11%)
Western Cape	63,226 (9%)	12,843 (9%)	76,069 (9%)
Mpumalanga	35,860 (5%)	5364 (4%)	41,224 (5%)
Free State	34,152 (5%)	10,642 (7%)	44,794 (5%)
North West	32,211 (5%)	5009 (3%)	37,220 (4%)
Limpopo	29,406 (4%)	6837 (5%)	36,243 (4%)
Northern cape	12,992 (2%)	2584 (2%)	15,576 (2%)
Missing	6282 (1%)	737 (1%)	7019 (2%)

### Validity of regression discontinuity design

Demographic and clinical characteristics were similar between patients just above and just below the log-standardized diagnosis threshold for both the type 2 and type 1 diabetes cohorts (Table 2).

**Table 2:** Predicted values of observed demographic characteristics of the type 2 and type 1 diabetes cohorts above and below the diagnostic threshold of diabetes.

**type 2 diabetes**			
	**Below the diagnostic threshold** **(n = 225,008; 32.8%)**	**Above the diagnostic threshold** **(n = 460,560; 67.2%)**	**P-value**
**Age (years)**	52.9	53.0	0.384
Missing, n (%)	0 (0%)	0 (0%)	
**Male (%)**	39.8%	39.8%	0.934
Missing, n (%)	0 (0%)	0 (0%)	
**First test location hospital**	66.4%	65.5%	0.099
Missing, n (%)	12,058 (5.4%)	18,366 (4.0%)	
**People Living with HIV**	18.1%	17.5%	0.206
Missing/unknown, n (%)	0 (0%)	0 (0%)	
**type 1 diabetes**			
	**Below the diagnostic threshold** **(n = 108,330; 76.6%)**	**Above the diagnostic threshold** **(n = 33,079; 23.4%)**	**P-value**
**Age (years)**	21.0	21.4	0.171
Missing, n (%)	0 (0%)	0 (0%)	
**Male (%)**	33.6%	33.6%	0.991
Missing, n (%)	0 (0%)	0 (0%)	
**First test location hospital**	84.9%	82.4%	0.011
Missing, n (%)	4,463 (4.1%)	1,145 (3.5%)	
**People Living with HIV**	17.2%	17.8%	0.562
Missing/unknown, n (%)	0 (0%)	0 (0%)	

The distribution of standardized diabetes test results was continuous over the threshold for both cohorts, with McCrary Density p-values of 0.3746 for the type 2 cohort (S1A Fig) and 0.1295 for the type 1 cohort (S1B Fig). These patterns did not suggest any manipulation of the assignment variable by either patients or providers; therefore, we believe the assumptions of the regression discontinuity method were met.

### Regression discontinuity results

#### Overall

Among patients in the type 2 diabetes cohort, 41.8% received a follow-up diabetes laboratory test within 24-months test after their initial screening test (S1 Table). This rate was slightly higher among those with a test result above the diagnostic threshold (52.4%; 184,334/351,928) (S1 Table). Among patients in the type 1 diabetes cohort, 24.6% received a follow-up test within 24-months after their initial screening test (S1 Table). This rate was 46.0% (10,041/21,817) among those with a diabetes test result above the diagnostic threshold (S1 Table).

**Fig 2:**
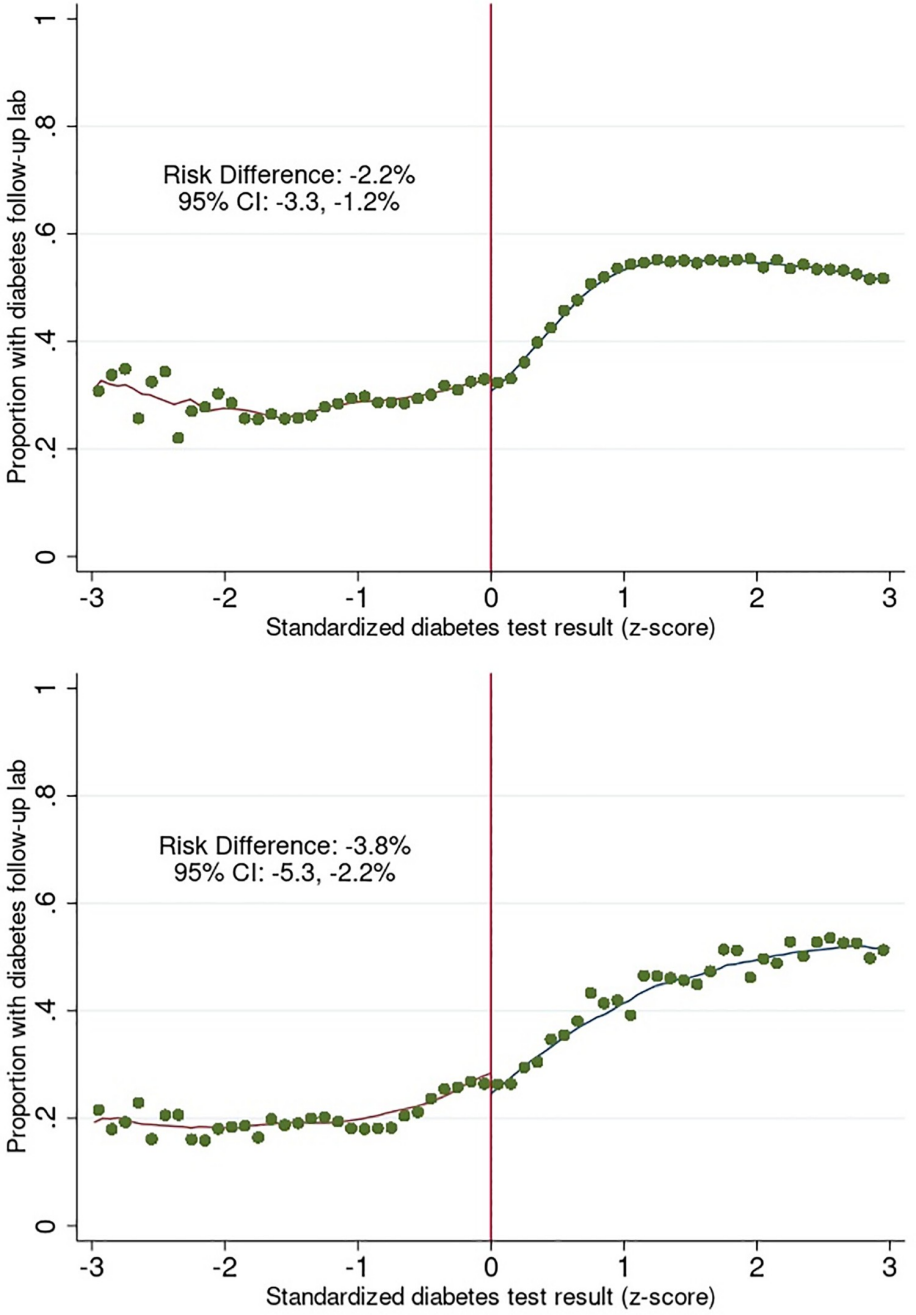
Probability of diabetes follow-up lab among a) type 2 cohort (adults aged >30 to 85 years) and b) type 1 cohort (adults aged 0 to <30 years).

The results of the regression discontinuity analysis are displayed in Fig 2. Within the optimal bandwidth imposed to achieve local randomization, patients in the type 2 diabetes cohort receiving a laboratory test result indicative of diabetes were 2.2% percentage points less likely to have a follow-up laboratory test compared to patients receiving a test result below the diagnostic threshold (risk difference (RD): -2.2%, 95% CI: -3.3%, -1.2%) (Fig 2A). Similar results were seen among patients in the type 1 diabetes cohort (RD: -3.8%, 95% CI: -5.3%, -2.2%) (Fig 2B). In both cohorts, trends on the right side of the threshold reveal that as the value of the diabetes test results increased, the probability of having a follow-up laboratory test also increased. Conversely, a similar pattern emerged on the left side of the diagnostic threshold, where lower diabetes test result values corresponded to a reduced likelihood of undergoing a follow-up test. However, these probabilities cannot be directly compared, as patients at the extreme ends of the spectrum may differ in both observed and unobserved characteristics.

#### Stratified by HIV status

**Fig 3:**
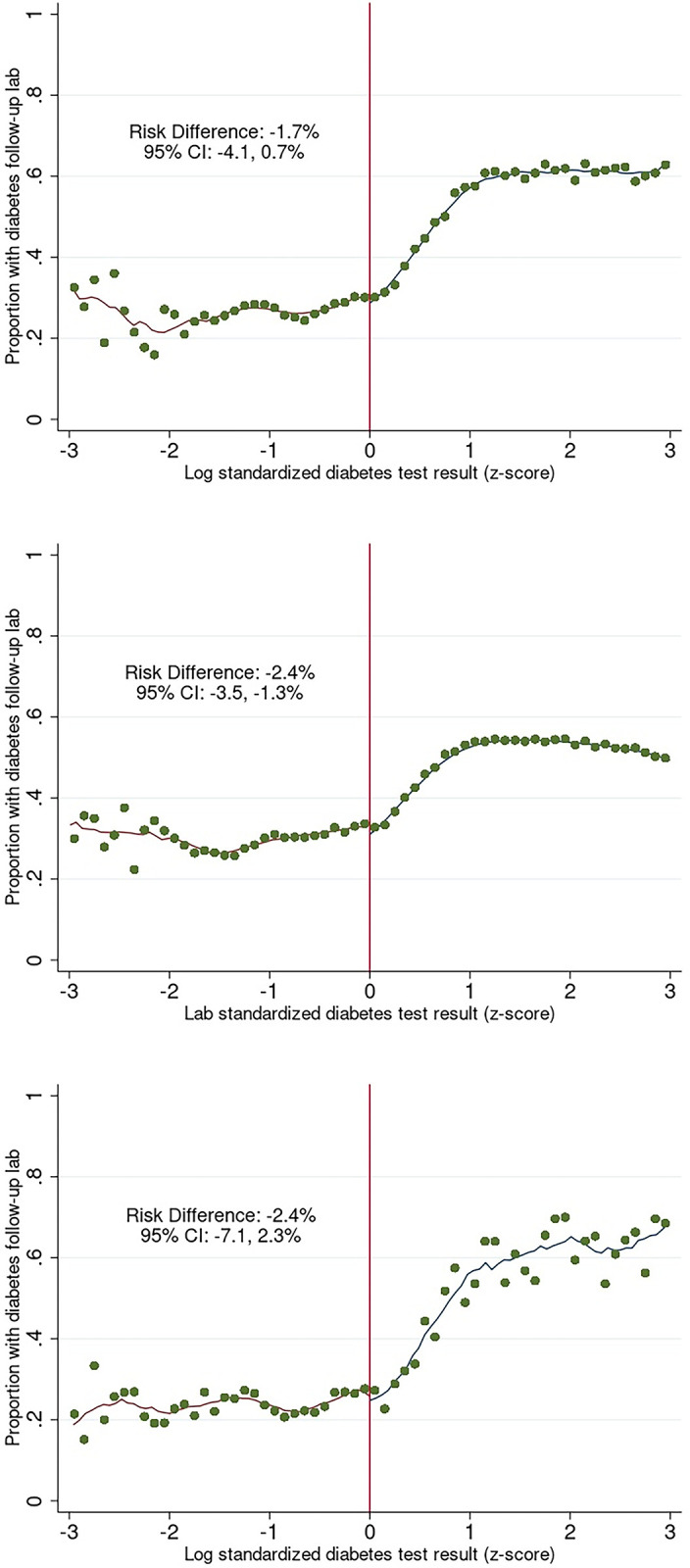
Probability of diabetes follow-up lab among a) type 2 cohort–people living with HIV, b) type 2 cohort–people living without HIV, c) type 1 cohort–people living with HIV, and d) type 1 cohort–people living without HIV.

When stratified by HIV status we found that among patients living with HIV in the type 2 diabetes cohort, 57.1% (n = 23,463/41,081) with a diabetes test result above the diagnostic threshold received a follow-up test within 24 months compared to 51.8% (158,650/305,976) of patients living without HIV. Among people living with HIV in the type 1 diabetes cohort, 56.8% (n = 1,820/3,205) received a follow-up test within 24 months compared to 44.4% (8089/18,221) of patients living without HIV. The results of the regression discontinuity analysis for both cohorts stratified by HIV status are displayed in Fig 3A and 3B. Findings again revealed no clinically meaningful difference in the likelihood of having a follow-up laboratory test within 24 months for either cohort. Similar to the overall results, trends on the right side of the threshold revealed that as diabetes test result values increased the probability of having a follow-up test also increased. This trend was particularly pronounced among people living with HIV in both cohorts (Fig 3A and 3C). We saw similar results when stratified by health facility setting (S2A–S2D Fig) for both cohorts.

#### Robustness checks

The robustness checks run on the crude (non-standardized) laboratory results data for HbA1c, fasting plasma glucose and random plasma glucose led to broadly similar results and did not change the substantive conclusions (S3A–S3F Fig). In all groups we saw essentially no difference in the likelihood of having a follow-up test within 24 months among patients with a test result just above versus below the diagnostic threshold. Estimates on the crude data were less precise, with the null value of 0 included in each 95% confidence interval.

## Discussion

The findings from our study underscore a concerning discrepancy in compliance to diabetes management guidelines within the South African healthcare system. Overall, follow-up rates for those that had a laboratory value indicative of diabetes were 46.0% (type 1 diabetes cohort) and 52.4% (type 2 diabetes cohort), suggesting that nearly half of patients with a laboratory test result above the diabetes diagnosis threshold are not receiving a follow-up test of glycemia, as stipulated by national diabetes management guidelines [5]. Even when considering patients with higher diabetes-related laboratory values, the proportion of individuals receiving follow-up laboratory tests did not exceed 60%, except for those living with HIV (type 1 diabetes cohort 69.6%; type 2 diabetes cohort 62.8%).

Ideally, a laboratory result above the diagnostic threshold for diabetes would trigger a follow-up laboratory test of glycemia within 3–6 months, in accordance with treatment guidelines [5]. Consequently, we expected to see a dramatic increase in the likelihood of receiving a follow-up laboratory test of glycemia at the diagnostic threshold. However, results of our analyses did not show this, suggesting limited compliance with laboratory-based decision rules. We understand that clinicians might prefer point-of-care testing over formal laboratory examinations for assessing glycemic control, given its immediate availability and quicker results. This could have led to some patients not having a follow-up test of glycemia recorded in the NHLS database. However, despite this tendency, a study of over 500,000 individuals attending public clinics in the Gauteng province during a similar period as our research found that point-of-care testing was used only 4% of the time, while formal laboratory tests accounted for 96% [18]. Our results could also be due to the challenges faced in resource-constrained settings where healthcare providers often need to prioritize care for patients with more acute medical needs and lack the necessary training to diagnose and treat chronic conditions [19]. In such contexts, healthcare providers might perceive patients whose laboratory results are in proximity to the diagnostic threshold as relatively healthy or less urgent cases. Another factor to consider is the patients’ capacity to attend a follow-up appointment at a healthcare facility. Even when healthcare services are offered free of charge, the monetary and time costs associated with traveling to a local clinic can present a significant barrier [20,21]. Moreover, despite the rising prevalence of diabetes in South Africa, there persists a deficiency in diabetes awareness at the level of the patient and the provider [22]. Consequently, for some patients, returning to the clinic for a follow-up test may not be a priority.

The limited follow-up rates and the lack of a clear distinction at the diagnostic threshold are especially worrisome for the type 1 diabetes cohort. Given that type 1 diabetes patients are generally more susceptible to complications and necessitate more rigorous monitoring due to the condition’s nature [22,23], one would anticipate a higher frequency of follow-up laboratory tests for this group. Yet, our observations align with prior research findings and do not show this expected trend [22,23]. Compromised glycemic control exposes individuals with type 1 diabetes to an elevated risk of early mortality. A two-decade-long study from Soweto, South Africa, revealed a staggering mortality rate of 43% among type 1 diabetes patients over 20 years [23]. Predominant causes of these untimely deaths were renal failure, hypoglycemia, and ketoacidosis [23].

Upon analyzing the outcomes among people living with HIV, a distinct pattern became evident. Patients living with HIV who displayed elevated test results demonstrated a marginally increased propensity to pursue additional laboratory tests (type 2 cohort 57.1%; type 1 cohort 56.6%). This suggests that individuals with a concurrent condition might receive closer surveillance and exhibit a higher inclination to follow through with subsequent testing recommendations. It is imperative to underscore that consistent and regular monitoring is essential for all individuals diagnosed with HIV. Those living with HIV encounter distinctive challenges in diabetes management, attributed to factors like chronic inflammation, insulin resistance, and potential antiretroviral drug side effects [24]. Chronic inflammation and insulin resistance, often observed in people living with HIV, can exacerbate glucose regulation, heightening their risk for diabetes-associated complications [25]. Moreover, certain antiretroviral medications can disrupt glucose balance, amplifying the importance of meticulous monitoring [26].

The primary strengths of our study include the extensive size of our national cohort (n = 826,977) and our employment of quasi-experimental techniques. Traditional epidemiological methods that utilize observational data to gauge compliance to guidelines and subsequent laboratory tests often suffer from confounding due to patient behaviors and are prone to selection bias. Our study mitigates some of these challenges by harnessing the regression discontinuity approach. Central to local randomization techniques is the premise that, within a regression discontinuity design, there is a specific bandwidth near the threshold where subjects appear as if randomly assigned to either the treatment or control group. By concentrating on those proximate to the diagnostic threshold, the only discernible distinction should hinge on whether a patient’s laboratory result falls above or below that threshold. This approach brings us nearer to simulating the conditions of a clinical trial, allowing for a more robust assessment of causal relationships.

Our study has some limitations. Missing data or instances where patients sought diabetes-related laboratory tests outside of the NHLS could introduce selection bias [27]. While efforts were made to link and validate data, such limitations are inherent in observational research [28]. Additionally, there may be clinical discretion as to whether a laboratory result above a certain value would be considered a diagnosis of diabetes and whether the provider feels a follow-up testis warranted. However, in our assessment of diabetes guidelines, we are limited to laboratory data only. Finally, though our analysis focused on the likelihood of having a follow-up laboratory test of glycemia, other aspects of diabetes management, such as treatment adherence and lifestyle modifications, were not considered [29].

## Conclusion

Our research sheds light on valuable opportunities for advancing diabetes care within the South African healthcare landscape and other resource-limited settings. There is an opportunity to reduce diabetes morbidity and mortality and achieve the WHO’s global targets [4] through increased follow-up of patients [30] and targeting of resources to patients with elevated laboratory test values, as indicated in national guidelines. The observed lack of follow-up rates among patients with a laboratory test result above the diabetes diagnosis threshold suggests that efforts should be directed towards implementing strategies that ensure timely and guideline-based follow-up care, especially for the type 1 diabetes cohort [31]. Moving forward, interventions that address barriers to guideline compliance and enhance patient education and healthcare provider communication, such as group counselling sessions and increasing the number of nurses available to treat NCDs like diabetes [32], are essential to minimize diabetes-related complications and optimize patient outcomes [33].

## Supporting information

S1 Fig Panel a) McCrary Density test of log-standardized diabetes test results to assess for data manipulation at the threshold of eligibility (bandwidth = 0.5, p-value = 0.3746) for the type 2 diabetes cohort; Panel b) McCrary Density test of log-standardized diabetes test results to assess for data manipulation at the threshold of eligibility (bandwidth = 0.5, p-value = 0.1295) for the type 1 diabetes cohort.
(DOCX)

S2 Fig Probability of diabetes follow-up lab in a) hospital type 2 cohort, b) PHC type 2 cohort, c) hospital type 1 cohort and d) PHC type 1 cohort.
(DOCX)

S3 Fig Robustness checks on crude lab data. Probability of diabetes follow-up lab among patients a) in the type 2 cohort whose first diabetes lab was a fasting glucose test, b) in the type 2 cohort whose first diabetes lab was an HbA1c test, c) in the type 2 cohort whose first diabetes lab was a random glucose test, d) in the type 1 cohort whose first diabetes lab was a fasting glucose test, e) in the type 1 cohort whose first diabetes lab was an HbA1c test, and f) in the type 1 cohort whose first diabetes lab was a random glucose test.
(DOCX)

S1 Table Proportion of patients receiving a follow-up lab within 24-months of first diabetes lab, stratified by test result of first diabetes lab. (DOCX)
