# Supplementary material for: Assessing compliance with national guidelines in diabetes care: A study leveraging data from south Africa’s National Health Laboratory Service (NHLS)
Source: PLOS Glob Public Health. 2024 Sep 3;4(9):e0003014. doi: 10.1371/journal.pgph.0003014 (PMC11371240; doi:10.1371/journal.pgph.0003014)
Supplement: S1 Fig — Panel a) McCrary Density test of log-standardized diabetes test results to assess for data manipulation at the threshold of eligibility (bandwidth = 0.5, p-value = 0.3746) for the type 2 diabetes cohort; Panel b) McCrary Density test of log-standardized diabetes test results to assess for data manipulation at the threshold of eligibility (bandwidth = 0.5, p-value = 0.1295) for the type 1 diabetes cohort. (DOCX) [file pgph.0003014.s001.docx]

|  |  |
| --- | --- |
| 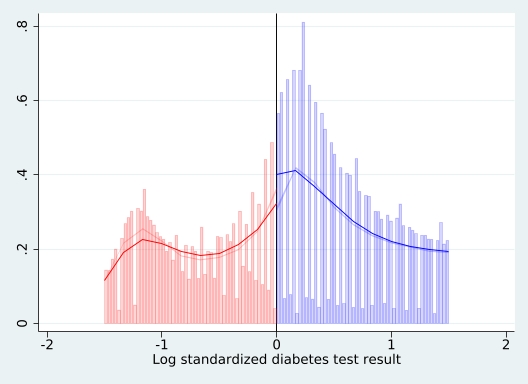 | 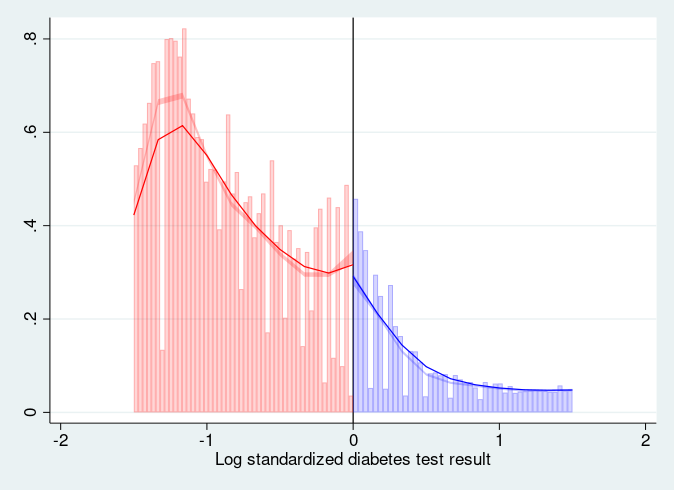 |

**S1 Fig**. Panel a) McCrary Density test of log-standardized diabetes test results to assess for data manipulation at the threshold of eligibility (bandwidth=0.5, p-value=0.3746) for the type 2 diabetes cohort; Panel b) McCrary Density test of log-standardized diabetes test results to assess for data manipulation at the threshold of eligibility (bandwidth=0.5, p-value=0.1295) for the type 1 diabetes cohort
