# Supplementary material for: Assessing compliance with national guidelines in diabetes care: A study leveraging data from south Africa’s National Health Laboratory Service (NHLS)
Source: PLOS Glob Public Health. 2024 Sep 3;4(9):e0003014. doi: 10.1371/journal.pgph.0003014 (PMC11371240; doi:10.1371/journal.pgph.0003014)
Supplement: S2 Fig — Probability of diabetes follow-up lab in a) hospital type 2 cohort, b) PHC type 2 cohort, c) hospital type 1 cohort and d) PHC type 1 cohort. (DOCX) [file pgph.0003014.s002.docx]

| a) Hospital (type 2 cohort) | b) Primary Health Centre (type 2 cohort) |
| --- | --- |
| 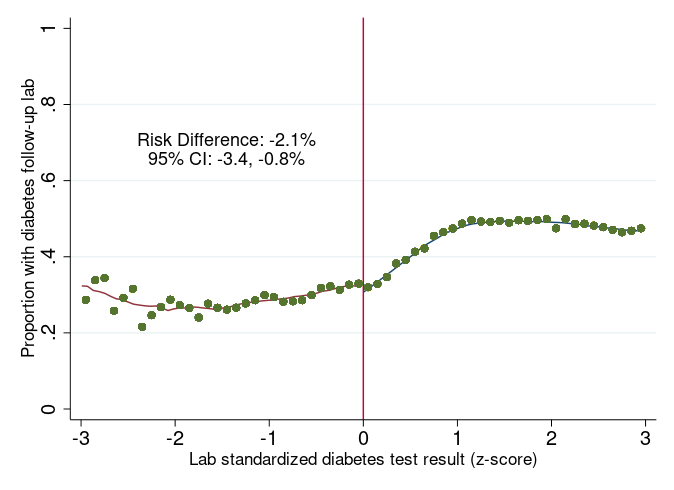 | 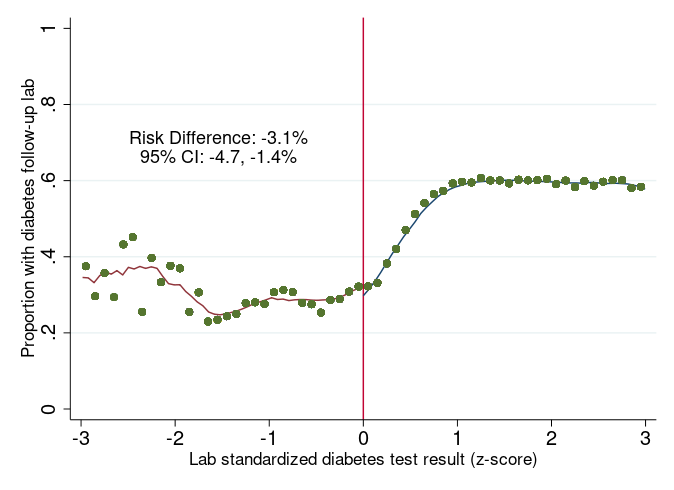 |
| c) Hospital (type 1 cohort) | d) Primary Health Centre (type 1 cohort) |
| 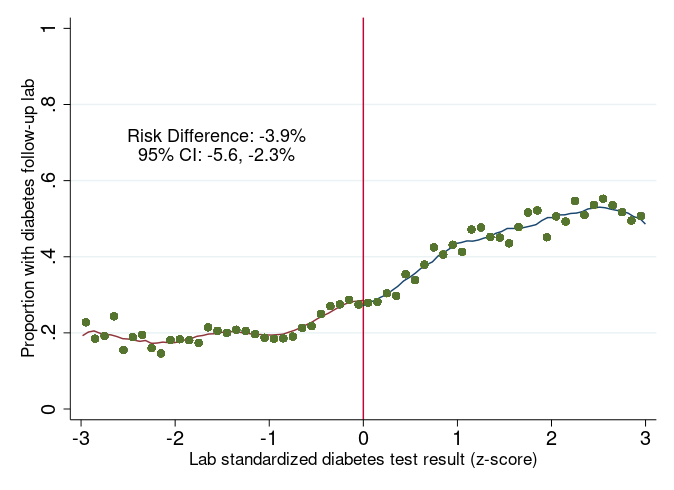 | 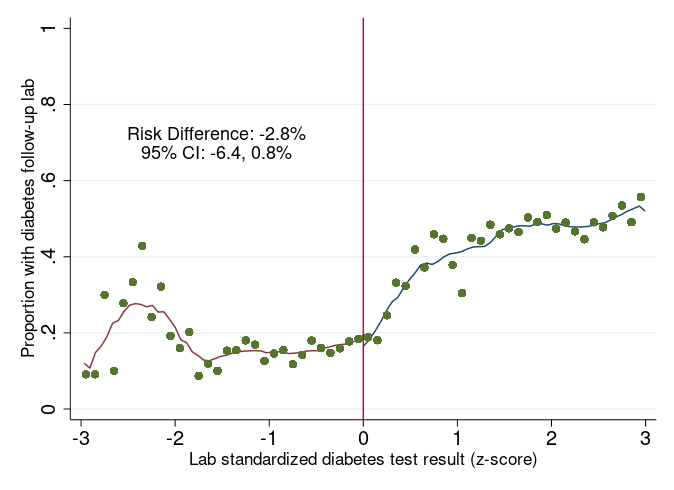 |

**S2 Fig** Probability of diabetes follow-up lab in a) hospital type 2 cohort, b) PHC type 2 cohort, c) hospital type 1 cohort and d) PHC type 1 cohort
