# Supplementary material for: Assessing compliance with national guidelines in diabetes care: A study leveraging data from south Africa’s National Health Laboratory Service (NHLS)
Source: PLOS Glob Public Health. 2024 Sep 3;4(9):e0003014. doi: 10.1371/journal.pgph.0003014 (PMC11371240; doi:10.1371/journal.pgph.0003014)
Supplement: S3 Fig — Probability of diabetes follow-up lab among patients a) in the type 2 cohort whose first diabetes lab was a fasting glucose test, b) in the type 2 cohort whose first diabetes lab was an HbA1c test, c) in the type 2 cohort whose first diabetes lab was a random glucose test, d) in the type 1 cohort whose first diabetes lab was a fasting glucose test, e) in the type 1 cohort whose first diabetes lab was an HbA1c test, and f) in the type 1 cohort whose first diabetes lab was a random glucose test. (DOCX) [file pgph.0003014.s003.docx]

| a) Fasting glucose (type 2 cohort) | b) HbA1c (type 2 cohort) |
| --- | --- |
| 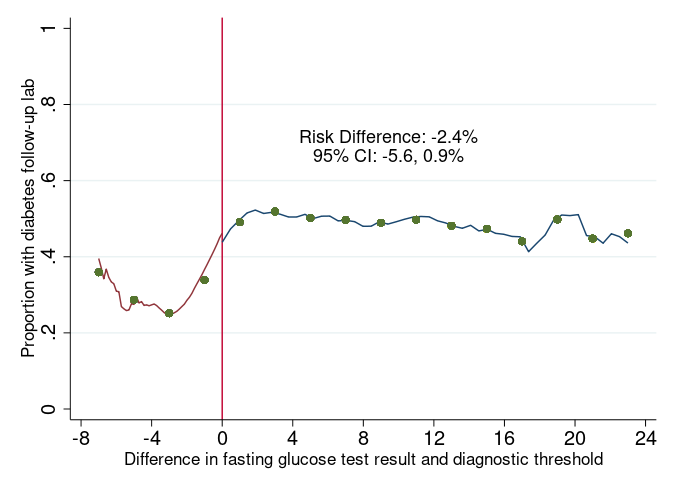 | 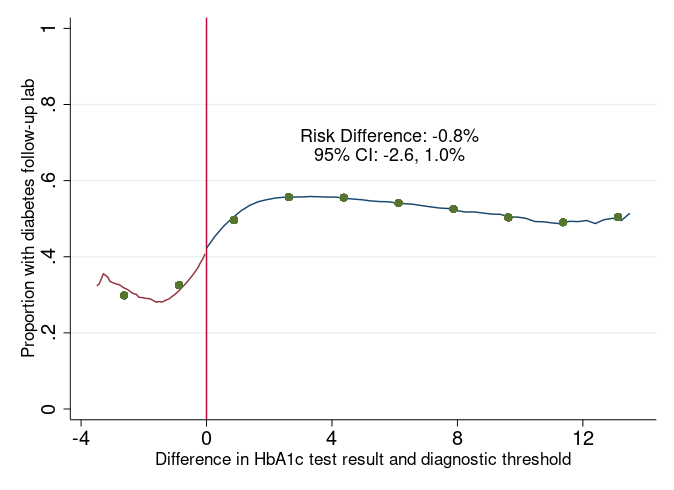 |
| c) Random glucose (type 2 cohort) | d) Fasting glucose (type 1 cohort) |
| 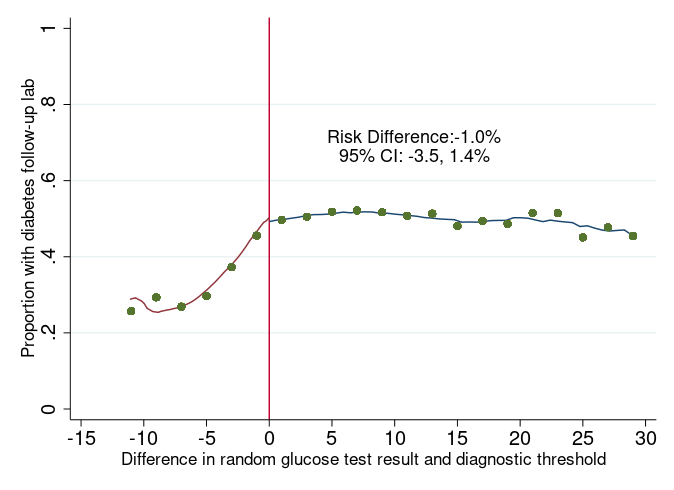 | 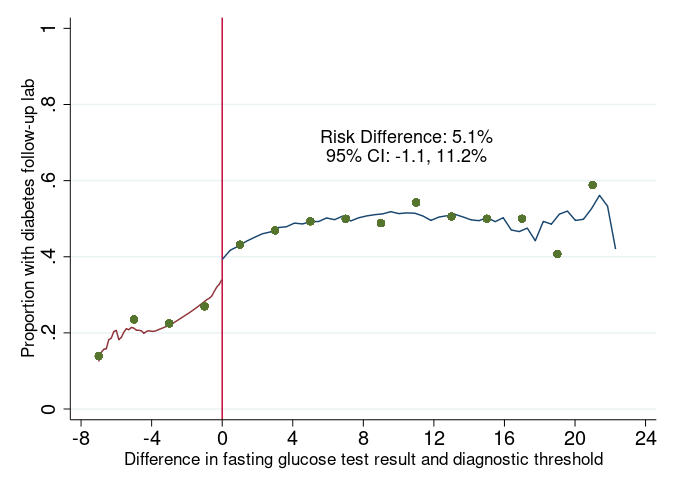 |
| e) HbA1c (type 1 cohort) | f) Random glucose (type 1 cohort) |
| 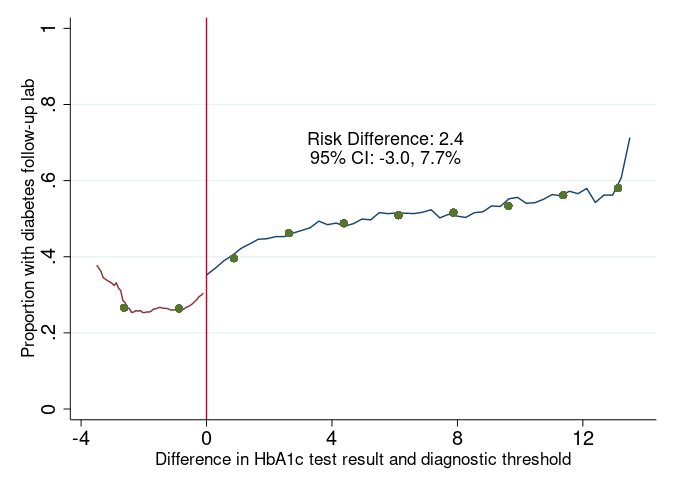 | 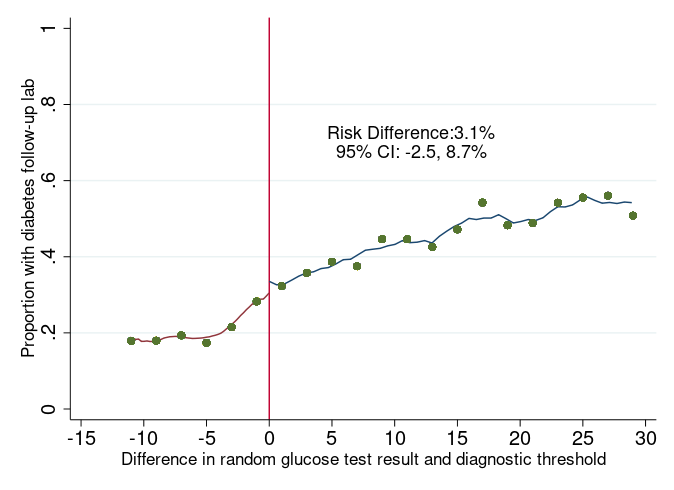 |

**S3 Fig.** Robustness checks on crude lab data. Probability of diabetes follow-up lab among patients a) in the type 2 cohort whose first diabetes lab was a fasting glucose test, b) in the type 2 cohort whose first diabetes lab was an HbA1c test, c) in the type 2 cohort whose first diabetes lab was a random glucose test, d) in the type 1 cohort whose first diabetes lab was a fasting glucose test, e) in the type 1 cohort whose first diabetes lab was an HbA1c test, and f) in the type 1 cohort whose first diabetes lab was a random glucose test
