# Supplementary material for: Assessing compliance with national guidelines in diabetes care: A study leveraging data from south Africa’s National Health Laboratory Service (NHLS)
Source: PLOS Glob Public Health. 2024 Sep 3;4(9):e0003014. doi: 10.1371/journal.pgph.0003014 (PMC11371240; doi:10.1371/journal.pgph.0003014)
Supplement: S1 Table — (DOCX) [file pgph.0003014.s004.docx]

**S1 Table.** Proportion of patients receiving a follow-up lab within 24-months of first diabetes lab, stratified by test result of first diabetes lab.

|  | **Type 2 Cohort** | | **Type 1 Cohort** | |
| --- | --- | --- | --- | --- |
|  | Test result below diagnostic threshold (n=343,801) | Test result above diagnostic threshold (n=351,928) | Test result below diagnostic threshold (n=122,452) | Test result above diagnostic threshold (n=21,817) |
| **Follow-up lab within 24 months** |  |  |  |  |
| Yes | 106,826 (31.1%) | 184,334 (52.4%) | 25,526 (20.9%) | 10,041 (46.0%) |
| No | 236,975 (68.9%) | 167,594 (47.6%) | 96,926 (29.1%) | 11,776 (54.0%) |
